# Elimination of schistosomiasis in China: Current status and future prospects

**DOI:** 10.1371/journal.pntd.0009578

**Published:** 2021-08-05

**Authors:** Wei Wang, Robert Bergquist, Charles H. King, Kun Yang

**Affiliations:** 1 Key Laboratory of National Health Commission of Parasitic Disease Prevention and Control, Jiangsu Provincial Key Laboratory of Parasites and Vector Control Technology, Jiangsu Institute of Parasitic Diseases, Wuxi, Jiangsu Province, China; 2 Ingerod, Brastad, Sweden (formerly with the UNICEF/UNDP/World Bank/WHO Special Programme for Research and Training in Tropical Diseases), World Health Organization, Geneva, Switzerland; 3 Center for Global Health and Diseases, Case Western Reserve University, Cleveland, Ohio, United States of America; 4 Center for Global Health, Nanjing Medical University, Nanjing, Jiangsu Province, China; Imperial College London, UNITED KINGDOM

## Abstract

Elimination of schistosomiasis as a public health problem among all disease-endemic countries in 2030 is an ambitious goal. Recent achievements resulting from mass drug administration (MDA) with praziquantel is promising but may need to be complemented with also other means. Schistosomiasis was highly prevalent in China before the initiation of the national schistosomiasis control program in the mid-1950s, and, at that time, the country bore the world’s highest burden of schistosomiasis. The concerted control efforts, upheld without interruption for more than a half century, have resulted in elimination of the disease as a public health problem in China as of 2015. Here, we describe the current status of schistosomiasis in China, analyze the potential challenges affecting schistosomiasis elimination, and propose the future research needs and priorities for the country, aiming to provide more universal insights into the structures needed for a global schistosomiasis elimination encompassing also other endemic regions.

## Introduction

Schistosomiasis, caused by blood flukes of the genus *Schistosoma* that depend on snail intermediate hosts for their life cycle, is a neglected tropical parasitic disease of great public health and socioeconomic significance [1]. This zoonotic parasitic disease is estimated to affect around 240 million people in 78 tropical and subtropical countries across the world, with up to 780 million at risk [2]. For more than 30 years, the World Health Organization (WHO) has recommended a morbidity control strategy through mass drug administration (MDA) with the drug praziquantel [3]. This approach has resulted in strong progress toward schistosomiasis elimination, as evidenced by a remarkable decline in prevalence and intensity of the disease [4,5]. This success provides hope that the agenda set for elimination of schistosomiasis in “this wormy world” has been correct [6]. However, the ambitious goal of eliminating the infection as a public health problem in all endemic countries in 2030 remains challenging [7], notably in African countries where more than 90% of global schistosomiasis patients live and where over 85% of local populations are constantly at risk [8,9]. This is partly because the current strategy for schistosomiasis control, despite the success mentioned above, fails to prevent reinfection. In addition, there are still inadequate supplies of praziquantel pills, which are primarily supplied through donations from international pharmaceutical companies [10–12].

The schistosome species in China is *Schistosoma japonicum*, and its snail host is *Oncomelania hupensis*, and the description of the disease caused by schistosomiasis dates back more than 2,200 years [13]. Significant *Schistosoma-*associated morbidity was highly prevalent before the initiation of the national schistosomiasis control program in the mid-1950s. Indeed, China had the highest burden of schistosomiasis in the world and at that time [14,15]. Since then, the strong political will [16], an effective and flexible control strategy at different program stages, and the rigorous surveillance and monitoring of factors associated with the transmission of schistosomiasis have resulted in elimination of the disease as a public health problem in China as of 2015 (Table 1) [17].

**Table 1:** Criteria for the elimination of schistosomiasis.

WHO criteria	Chinese criteria
**•** **Disease control**: prevalence of heavy-intensity infection <5% in all schistosomiasis-endemic setting of the country; **•** **Elimination of schistosomiasis as a public health problem**: prevalence of heavy-intensity infection <1% in all schistosomiasis-endemic setting of the country; **•** **Elimination of transmission**: reduction of infection incidence to zero.	**•** **Infection control**: prevalence of infection <5% in humans and livestock and absence of outbreaks of acute schistosomiasis; **•** **Transmission control**: prevalence of infection <1% in humans and livestock, including absence of both (i) local *O*. *hupensis* infected by *S*. *japonicum* (sampled for 2 consecutive days); and (ii) any local cases of acute schistosomiasis; **•** **Transmission interruption**: no *S*. *japonicum* infections identified in local residents or livestock for 5 consecutive years and absence of *O*. *hupensis* infected by *S*. *japonicum* for at least 5 years; **•** **Elimination**: absence of *S*. *japonicum* in local residents, livestock, or in *O*. *hupensis* for subsequent 5 years after transmission interruption.

WHO, World Health Organization.

As can be seen in Table 1, the Chinese criteria regarding schistosomiasis elimination are even more stringent than those recommended by WHO [18]. The country remains currently at the “pre-elimination” stage [19], and we are aware that there are still challenges that may hinder the progress toward final disease elimination [20,21]. Here, we describe the current status of schistosomiasis in China, analyze the potential challenges affecting elimination, and propose the future research needs and priorities for the country. This discussion is also intended to provide more universal insights into the structures needed for a global schistosomiasis elimination program encompassing all endemic regions of the world.

## Elimination of schistosomiasis in China: Current status

**Fig 1:**
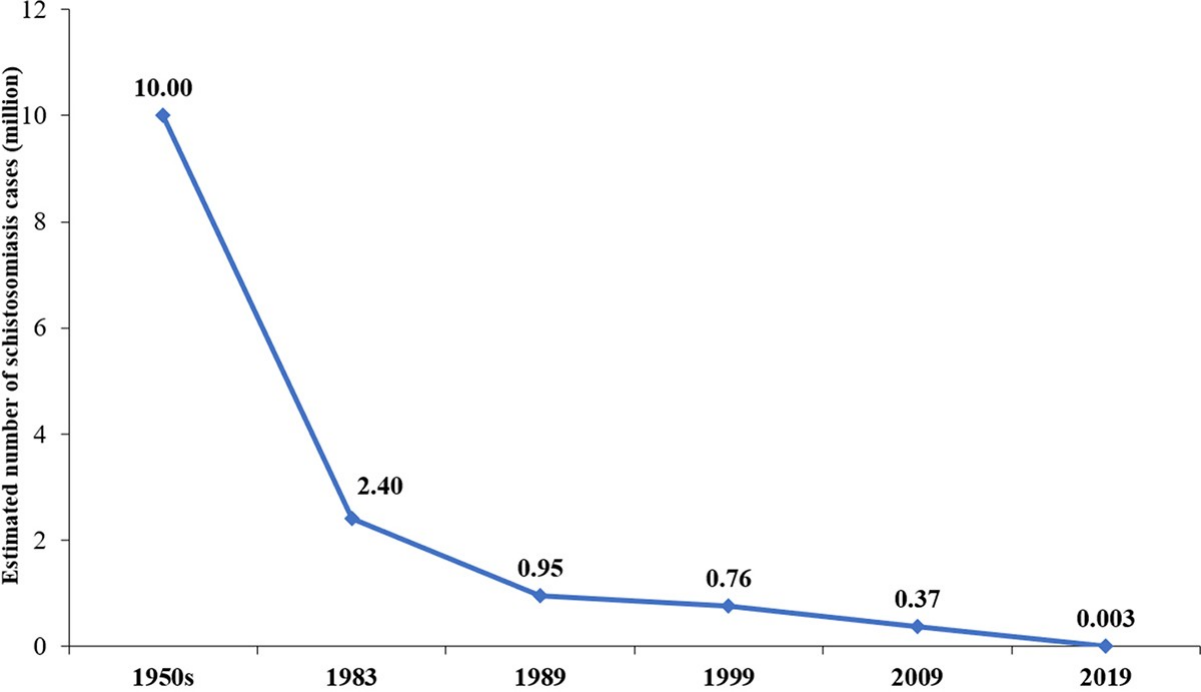
Changes in the estimated number of schistosomiasis cases in China from 1950s to 2019.

**Fig 2:**
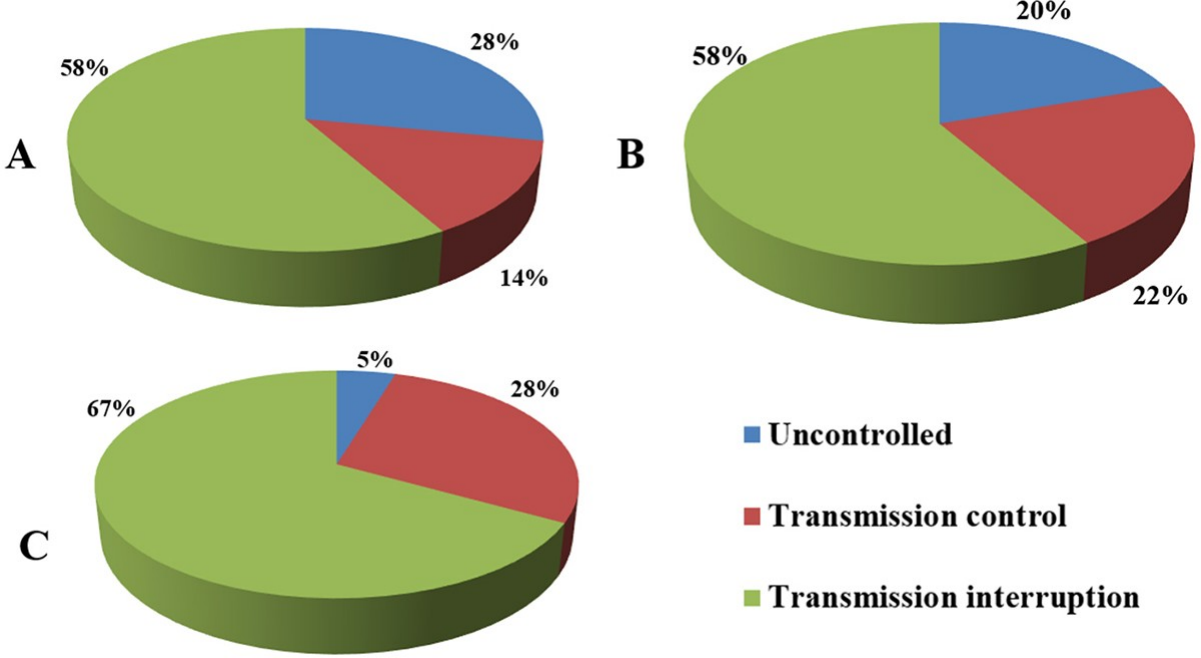
The changing proportions of infection control categories among schistosomiasis-endemic counties in different periods of schistosomiasis control in China from 1999 to 2019: **(A)** 1999, **(B)** 2009, and **(C)** 2019.

**Fig 3:**
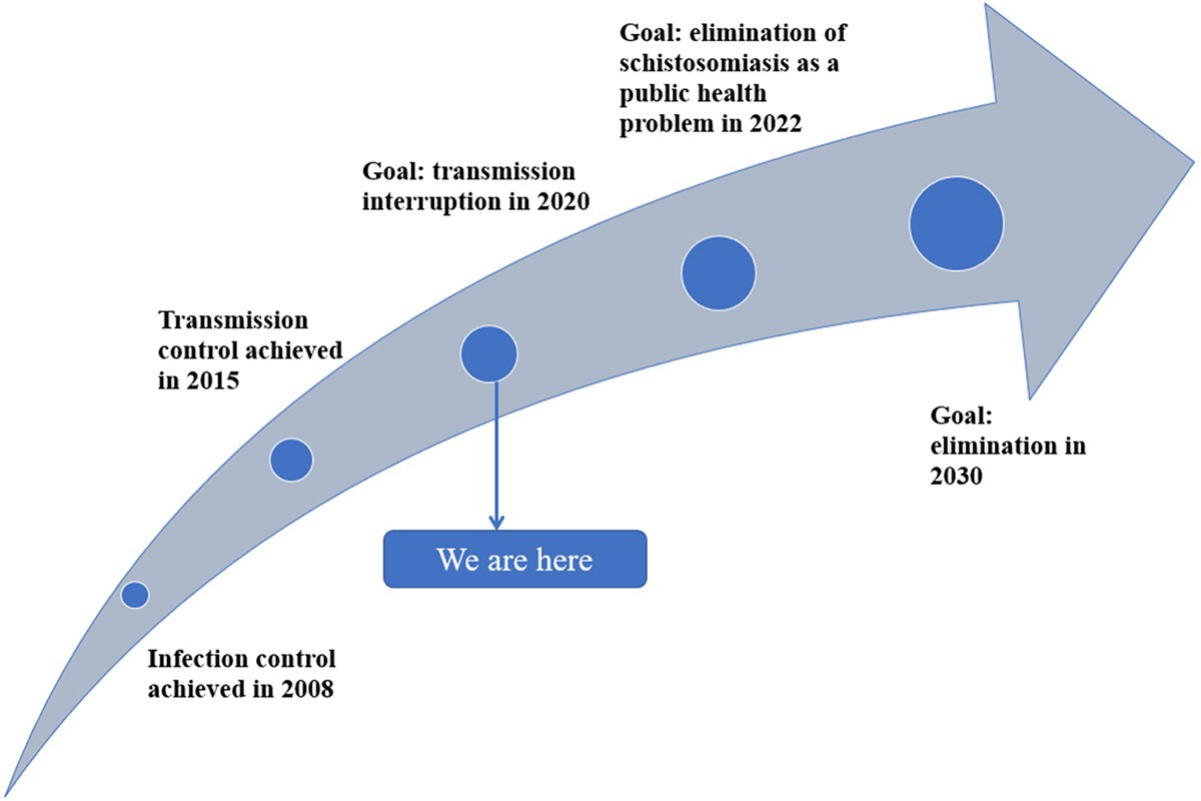
Overall progress of schistosomiasis elimination in the country.

Following China’s concerted effort to control and eliminate schistosomiasis that has been ongoing for nearly 70 years [1], the endemic status is currently characterized by an extremely low prevalence and intensity of *S*. *japonicum* infections [22]. In 2019, only 30,000 individuals were estimated to have the disease across the country, with only 5 new infections detected that year [23]. This indicates a more than 99% reduction in the number of cases as compared to the situation in the 1950s (Fig 1) [23–27]. In addition, in 2019, the transmission of schistosomiasis had been controlled, or interrupted in approximately 95% of the 450 counties that were once considered endemic for *S*. *japonicum*, including 67 that have now achieved elimination (Figs 2 and 3). These data demonstrate that we are steadily approaching to final demise of the “God of Plague” [17]. It has been well known that experiences from the great success in schistosomiasis control in China include high-level political sustained commitment [28], persistent and adequate financial support [29], and integrated control mobilizing multi-sectoral resources [30,31]. Nevertheless, the following approaches have also contributed to this success: (1) government-led containment: In China, the national schistosomiasis control program is included in the annual governmental work plan at national, provincial, municipal, county, and township levels, and all schistosomiasis control approaches are implemented under governmental leadership, ensuring the smooth implementation of all activities [16]; (2) nationwide participation: All residents living in schistosomiasis-endemic foci are encouraged to participate in the schistosomiasis control program [30]; (3) a unique control strategy: Although there have been 3 major shifts in the national schistosomiasis control strategy, a unique strategy is employed across the whole country [28]; and (4) a powerful surveillance-response system: The strong monitoring capability and rapid response mechanism allows effective management of schistosomiasis epidemics, making eradication of the transmission risk possible [28].

### Challenges

Although China is moving steadily toward complete elimination of schistosomiasis across the country, many challenges remain in order to eliminate this disease by 2030 (Table 2) [32]. Among them is financial support, the most urgent issue that must always be addressed. Indeed, one of the important lessons learned from the World Bank Loan Project (WBLP) for Chinese Schistosomiasis Control Program (1992 to 2001) is that sustained financial support is needed for effective program performance. The absence of adequate support after the end of the WBLP resulted in rebound of schistosomiasis transmission [29]. Since all interventions included in the national schistosomiasis elimination program are fully paid by the government and that reduced funding would fail to support the integrated control interventions and capability improvements agreed, it is essential that strong political support continues along with provision of commensurate financial support to maintain China’s elimination initiative.

**Table 2:** Potential challenges for elimination of schistosomiasis in China.

Points of concern	Main challenge
**•** Financial support	Since the central government took over responsibility for the financial expenditure of the national schistosomiasis control program in 2018, local governments decreased or even ceased their support. This may make it difficult to sustain current control activities in the face of the continuous rise of the cost of products used for schistosomiasis elimination, manpower resources, and training.
**•** Snail control	Increasing rigorous environmental protection policies restrict the use of chemical snail control, which particularly affects activities in the endemic areas in the lake and mountainous regions.
**•** Emerging and reemerging sources of infections	Emerging *S*. *japonicum* reservoir hosts, such as goats, canines, and wild mice, are neglected but play an important role in the transmission of the parasite. In addition, protection of endangered species of deer, such as *E*. *davidianus*, poses a threat to continued control in certain places.
**•** Frequent natural disasters	Frequent flooding along the Yangtze River basin, the main schistosomiasis-endemic focus of China, and earthquakes can lead to alterations of snail-infested settings, resulting in snails to be spread into new areas. In addition, individuals infected with *S*. *japonicum* infections may be displaced into new transmission zones due to such events.
**•** Capability building	Grassroot staff remain the main force in the operation of the schistosomiasis elimination program; however, these workers currently suffer from problems related to old age, low education level, and minimum salaries, all of which have led to a high proportion of resignations, resulting in a reduced schistosomiasis control capability. In addition, governmental institution reforms have reduced the number of workers participating in schistosomiasis control, so many now work only part time. The reduced financial support further hinders the updating of hardware and restricts training of new control professionals.
**•** Integrated control	One of the most important lessons learned from the Chinese National Schistosomiasis Control Program is the need for mobilization of multi-sectoral resources and use of multiple interventions in an integrated manner; however, lack of sufficient financial support restricts collaboration of the health sector with the agriculture, water conservancy, forestry, and education sectors.
**•** Imported schistosomiasis	China’s policy of opening up, and its “Belt and Road” initiative in Africa, has recently resulted in imported cases of *Schistosoma mansoni* and *Schistosoma haematobium* infections not infrequently found also in China. Given the existence of *Biomphalaria straminea* in southern China and its capability of acting as intermediate host snail for *S*. *mansoni*, there is a growing concern about potential *S*. *mansoni* transmission in mainland China. In addition, the potential hybridization between *S*. *japonicum* and *S*. *mansoni* may affect the progress of the elimination program.
**•** Urban schistosomiasis	Schistosomiasis predominantly occurs in rural and poor regions. However, with the acceleration of China’s urbanization, the boundary between urban and rural regions has become increasingly unclear. In addition, due to the introduction of intermediate snail hosts resulting from construction of urban wetlands, the transmission of *S*. *japonicum* is more and more likely in urban regions.

**Fig 4:**
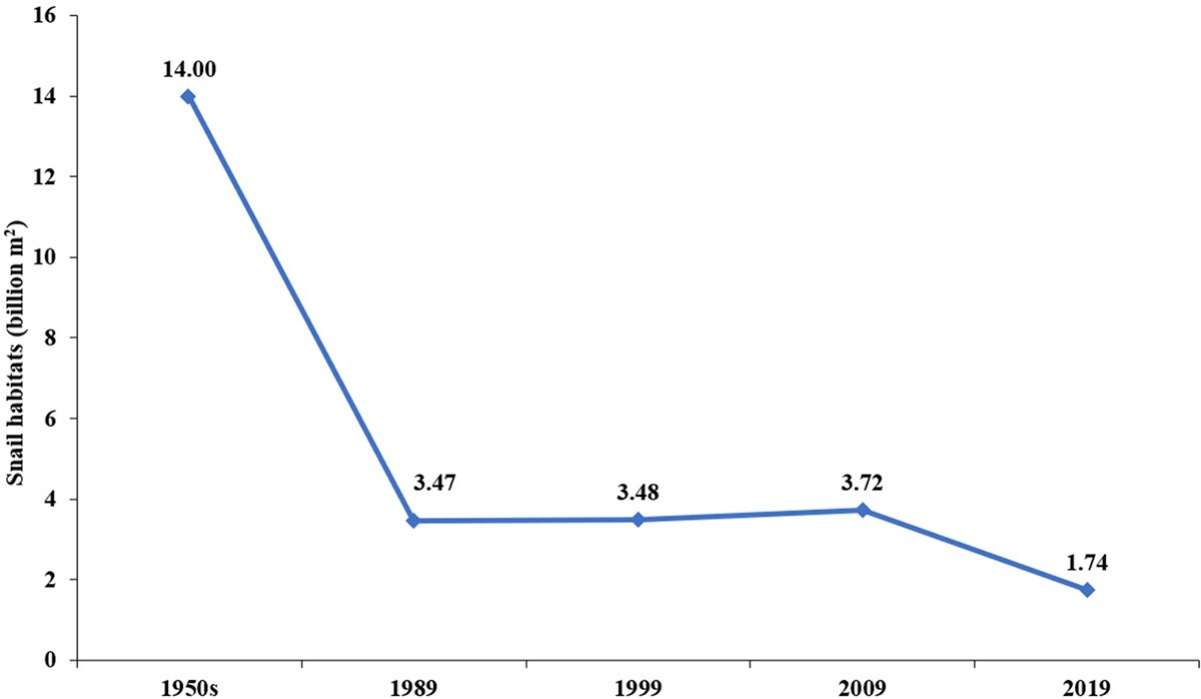
Changes in the area of *O*. *hupensis* snail habitats in China from 1950s to 2019.

Snail control has proven to be one of the most effective tools for interruption of schistosomiasis [33–35], particularly with regard to *S*. *japonicum*, as its snail host is amphibious, and, therefore, more vulnerable than its African counterparts. This approach has been an important part of the Chinese national schistosomiasis control strategy since the 1950s (Fig 4) [36]. Although no *S*. *japonicum* infections have been identified in *O*. *hupensis* snails since 2014 [37], 1.74 billion m^2^ snail habitats were detected across China in 2019, including 642,000 m^2^ emerging snail-infested settings and 8.51 million m^2^ reemerging settings [23]. These widespread host snails are an important unknown factor during the stage when we move toward elimination of schistosomiasis.

An unfortunate duality with respect to the need for snail control has arisen from the government’s call for environmental protection, which restricts the use of chemical treatment as part of snail control, while the development of China’s Yangtze River Economic Belt prohibits the use of environmental change, such as dams for snail control, along the Yangtze River basin—the main schistosomiasis-endemic focus of China. Meanwhile, the construction of large-scale wetlands has led to the introduction of *O*. *hupensis* snails into urban areas [38]. Agricultural developments, water conservancy, and deforestation programs have shown feasible for the improving snail-infested settings; however, these programs require costly investments [31,39]. To add fuel to the fire, the termination of financial support given to agriculture, water conservancy, and forestry sectors for schistosomiasis control programs since 2016 has resulted in ineffective multi-sectoral collaborations and cessation of environmental improvements for snail control. In addition, frequent floods along the Yangtze River basin [40–42] and earthquakes in the mountainous schistosomiasis-endemic areas may now lead to the spread of *O*. *hupensis* snails and expansion of schistosomiasis transmission as a result [43–45].

Unlike the other 2 main species of the genus *Schistosoma* infective to humans, *S*. *japonicum* is a zoonotic parasite with over 40 different mammalian species serving as reservoir hosts [46]. Although bovines are included in the Chinese National Schistosomiasis Control Program and given high priority for control interventions as a major source of infection [47], goats, dogs, and wild mice, which are also reservoirs for *S*. *japonicum*, are generally not targeted [48]. Previous studies have shown that these animals also play an increasingly important role in the transmission of schistosomiasis [20,49]. A recent meta-analysis showed 3.9% overall pooled prevalence of *S*. *japonicum* infections in 8,795 wild rodents based on 37 eligible publications, involving 61 field studies across mainland China from 1980 to 2020 [20]. No comparable overall change of the infection prevalence was detected in rodents with great reductions in the numbers of schistosomiasis cases in humans and bovines; however, importantly, there appeared to be an increase in *S*. *japonicum* prevalence in rodents over time within the hilly and mountainous endemic regions [20]. Since rodents were projected to become the dominant wildlife in human-driven environments and the main reservoir of zoonotic diseases in general within tropical zones, intensified monitoring and evaluation of potential *S*. *japonicum* infections within rodents is strongly recommended, particularly in hilly and mountainous regions [20]. A very recent, preprint publication also reports high prevalence of *Schistosoma* infections in rodents, highlighting the important role of rodents in schistosomiasis transmission across the world during the current stage of disease elimination [50]. Moreover, *Elaphurus davidianus*, a type of deer native to the river valleys of China, is a Grade 1 state-protected animal in China that can be seen grazing in marshlands around Poyang Lake and Dongting Lake areas, which are places where *O*. *hupensis* snails infected by *S*. *japonicum* have previously been found with high prevalence and intensity of infection [51–53]. In 2004, a field investigation detected 29.2% (38/130) of the wild-captured *E*. *davidianus* fecal samples positive for *S*. *japonicum* in Gudao section of the Yangtze River in Shishou City, central China [53]. In 2016, 16% (4/25) of the wild *E*. *davidianus* fecal samples captured from the Tian’e marshland of Shishou City were identified as infected by *S*. *japonicum* [52]. A recent study shows a 4.4% (2/46) detection of *S*. *japonicum* eggs in stool samples of *E*. *davidianus* captured from the grassland of Poyang Lake with a high intensity of infection based on the miracidial hatching test (+++) found [51]. This abundance of these deer poses a high risk of them becoming an emerging reservoir of *S*. *japonicum* infections, in particular since it is prohibited to investigate this animal or provide any kind of chemotherapy if needed. What makes matters worse is that fencing is also not allowed, while free grazing in the grasslands is encouraged [54]. Thus, the presence of this type of deer in areas still endemic for schistosomiasis is a big threat with respect to consolidation of the achievements gained from current concerted control efforts, and it endangers the progress toward elimination of schistosomiasis in China.

## Schistosomiasis elimination in China: Future requirements

The experience of many global schistosomiasis control programs has shown that MDA with praziquantel alone is unlikely to eliminate schistosomiasis [55], and an integrated strategy mobilizing resources from health, agriculture, water conservancy, forestry, and education sectors is required [56]. To tackle the challenges mentioned above, the complete eradication of potential risks for resurgence of the disease is of great and urgent need. During the stage of moving toward elimination of schistosomiasis, a more fine-grained precision control is recommended as the choice for managing interventions as part of the national schistosomiasis elimination program (Table 3) [57].

**Table 3:** Future research priorities for the elimination of schistosomiasis in China.

Target	Intervention
**•** Precision identification	➢ Identification of high-risk settings using geospatial tools; ➢ Monitoring of domestic and wild animals in snail-infested marshlands using AI and unmanned aerial vehicles; ➢ Assessing the role of reservoir hosts in the local transmission of schistosomiasis using epidemiological tools; ➢ Development of assays for early and sensitive detection of *S*. *japonicum* infections in humans, livestock, and *O*. *hupensis* snails; ➢ Identification and tracking of high-risk populations using high-level internet connection and approaches based on machine learning.
**•** Precision treatments	➢ Development of health education materials based on social media, such as “WeChat”; ➢ Development of highly effective, minimally toxic, and environmentally friendly molluscicides; ➢ Development of orally administered agents for prevention of infections; ➢ Establishing a smart system to achieve real-time monitoring of schistosomiasis control data using cell phone technology; ➢ Building a sensitive and effective surveillance and forecast system as a response to emergency outbreaks and imported epidemics.
**•** Precision evaluations	➢ Assessing the performance of praziquantel chemotherapy with regard to local transmission of schistosomiasis in order to avoid overtreatment; ➢ Assessing the performance of chemical treatment with regard to local transmission of schistosomiasis to avoid over-mollusciciding; ➢ Assessing the cost-effectiveness and cost-benefit of different packaging interventions to adjust optimal intervention combinations adapted to local epidemiological features and socioeconomic developments.

AI, artificial intelligence.

## Discussion and conclusions

Rapid social and economic development during the past decades has brought with it striking achievements in the Chinese National Schistosomiasis Control Program. Remarkably, in 2019, only 5 individuals tested positive for *S*. *japonicum* throughout the country [23]. Currently, the transmission of schistosomiasis is almost interrupted, and the country is steadily moving toward schistosomiasis elimination. In addition to the integrated strategy [58], Chinese experience and tools are now being transferred overseas and have been applied in countries affected by schistosomiasis in Africa and Southeast Asia [59–62]. It is clear that the experience gained and lessons learned over the past 7 decades of schistosomiasis control in China can provide valuable insights useful for the ongoing global schistosomiasis elimination program, as outlined in the new WHO Roadmap for Neglected Tropical Diseases [7].

## References

[1] Colley DG, Bustinduy AL, Secor WE, King CH. Human schistosomiasis. *Lancet*. 2014;383(9936):2253–64. doi: 10.1016/S0140-6736(13)61949-2 24698483 PMC4672382

[2] McManus DP, Dunne DW, Sacko M, Utzinger J, Vennervald BJ, Zhou XN. Schistosomiasis. *Nat Rev Dis Primers*. 2018;4(1):13. doi: 10.1038/s41572-018-0013-8 30093684

[3] WHO. Prevention and control of schistosomiasis and soil-transmitted helminthiasis: WHO Technical Report Series N° 912. Report of a WHO expert committee. Geneva: WHO; 2002. 12592987

[4] Mutapi F, Maizels R, Fenwick A, Woolhouse M. Human schistosomiasis in the post mass drug administration era. *Lancet Infect Dis*. 2017;17(2):e42–8. doi: 10.1016/S1473-3099(16)30475-3 27988094 PMC7614913

[5] GBD 2017 Disease and Injury Incidence and Prevalence Collaborators. Global, regional, and national incidence, prevalence, and years lived with disability for 354 diseases and injuries for 195 countries and territories, 1990–2017: a systematic analysis for the Global Burden of Disease Study 2017. *Lancet*. 2018; 392(10159): 1789–1858. doi: 10.1016/S0140-6736(18)32279-7 30496104 PMC6227754

[6] Deol AK, Fleming FM, Calvo-Urbano B, Walker M, Bucumi V, Gnandou I, et al. Schistosomiasis—Assessing progress toward the 2020 and 2025 global goals. *N Engl J Med*. 2019;381(26):2519–28. doi: 10.1056/NEJMoa1812165 31881138 PMC6785807

[7] WHO. Ending the neglect to attain the Sustainable Development Goals: a road map for neglected tropical diseases 2021–2030. Geneva: World Health Organization; 2020 [cited 2020 Oct 21]. Available from: https://www.who.int/neglected_diseases/WHONTD-roadmap-2030/en/

[8] Lai YS, Biedermann P, Ekpo UF, Garba A, Mathieu E, Midzi N, et al. Spatial distribution of schistosomiasis and treatment needs in sub-Saharan Africa: a systematic review and geostatistical analysis. *Lancet Infect Dis*. 2015;15(8):927–40. doi: 10.1016/S1473-3099(15)00066-3 26004859

[9] Hotez PJ, Fenwick A. Schistosomiasis in Africa: an emerging tragedy in our new global health decade. *PLoS Negl Trop Dis*. 2009;3(9):e485. doi: 10.1371/journal.pntd.0000485 19787054 PMC2746322

[10] Wang W, Liang Y. Mass drug administration (MDA) for schistosomiasis. *J Infect Dis*. 2015;211(5):848–9. doi: 10.1093/infdis/jiu506 25205633

[11] Lo NC, Addiss DG, Hotez PJ, King CH, Stothard JR, Evans DS, et al. A call to strengthen the global strategy against schistosomiasis and soil-transmitted helminthiasis: the time is now. *Lancet Infect Dis*. 2017;17(2):e64–9. doi: 10.1016/S1473-3099(16)30535-7 27914852 PMC5280090

[12] Tchuem Tchuenté LA, Rollinson D, Stothard JR, Molyneux D. Moving from control to elimination of schistosomiasis in sub-Saharan Africa: time to change and adapt strategies. *Infect Dis Poverty*. 2017;6(1):42. doi: 10.1186/s40249-017-0256-8 28219412 PMC5319063

[13] Mao SP, Shao BR. Schistosomiasis control in the People’s Republic of China. *Am J Trop Med Hyg*. 1982;31(1):92–9. 7058983

[14] Chen MG. Progress and problems in schistosomiasis control in China. *Trop Med Parasitol*. 1989;40(2):174–6. 2505378

[15] Song LG, Wu XY, Sacko M, Wu ZD. History of schistosomiasis epidemiology, current status, and challenges in China: on the road to schistosomiasis elimination. *Parasitol Res*. 2016;115(11):4071–81. doi: 10.1007/s00436-016-5253-5 27679451

[16] Chen J, Xu J, Bergquist R, Li SZ. Zhou XN. "Farewell to the God of Plague": The importance of political commitment towards the elimination of schistosomiasis. *Trop Med Infect Dis*. 2018;3(4):108. 10.3390/tropicalmed3040108 PMC6306784 30282897

[17] Xu J, Li SZ, Zhang LJ, Bergquist R, Dang H, Wang Q, et al. Surveillance-based evidence: elimination of schistosomiasis as a public health problem in the Peoples’ Republic of China. *Infect Dis Poverty*. 2020;9(1):63. doi: 10.1186/s40249-020-00676-5 32505216 PMC7275476

[18] Ministry of Health. Control and elimination of schistosomiasis. Beijing: China Standard Publishing House, 2016; 1–4 (in Chinese).

[19] Cao CL, Zhang LJ, Deng WP, Li YL, Lv C, Dai SM, et al. Contributions and achievements on schistosomiasis control and elimination in China by NIPD-CTDR. *Adv Parasitol*. 2020;110:1–62. doi: 10.1016/bs.apar.2020.04.002 32563322

[20] Zou HY, Yu QF, Qiu C, Webster JP, Lu DB. Meta-analyses of *Schistosoma japonicum* infections in wild rodents across China over time indicates a potential challenge to the 2030 elimination targets. *PLoS Negl Trop Dis*. 2020;14(9):e0008652. doi: 10.1371/journal.pntd.0008652 32877407 PMC7491725

[21] Jing X, Shan L, Chun-Li C, Shi-Zhu L, Xiao-Nong Z. Progress and challenges of schistosomiasis elimination in China. *Zhongguo Xue Xi Chong Bing Fang Zhi Za Zhi*. 2018;30(6):605–9. (in Chinese). doi: 10.16250/j.32.1374.2018249 30891968

[22] Guo JY, Xu J, Zhang LJ, Lv S, Cao CL, Li SZ, et al. Surveillance on schistosomiasis in five provincial-level administrative divisions of the People’s Republic of China in the post-elimination era. *Infect Dis Poverty*. 2020;9(1):136. doi: 10.1186/s40249-020-00758-4 33004080 PMC7528395

[23] Zhang LJ, Xu ZM, Dang H, Li YL, Lü S, Xu J, et al. Endemic status of schistosomiasis in People’s Republic of China in 2019. *Zhongguo Xue Xi Chong Bing Fang Zhi Za Zhi*.: 2020;32(6):551–558. (in Chinese). doi: 10.16250/j.32.1374.2020263 33325187

[24] Basch PF. Schistosomiasis in China: an update. *Am J Chin Med*. 1986;14(1–2):17–25. doi: 10.1142/S0192415X86000041 3083670

[25] Cen LP. A short review of the previous and current epidemiological situation of schistosomiasis in China. *Rev Soc Bras Med Trop*. 1997;30(1):57–60. doi: 10.1590/s0037-86821997000100011 8993107

[26] Wang LY, Jiang QW, Liu JX, Zhao GM, Chen XY. Schistosomiasis situation in People’s Republic of China in 1999. *Zhongguo Xue Xi Chong Bing Fang Zhi Za Zhi*. 2000;12(6):321–3. (in Chinese). 23593826

[27] Hao Y, Zheng H, Zhu R, Guo JG, Wang LY, Chen Z, et al. Schistosomiasis situation in People’s Republic of China in 2009. *Zhongguo Xue Xi Chong Bing Fang Zhi Za Zhi*. 2010;12(6):521–7. (in Chinese). 23593826

[28] Wang L, Utzinger J, Zhou XN. Schistosomiasis control: experiences and lessons from China. *Lancet*. 2008;372(9652):1793–935. doi: 10.1016/S0140-6736(08)61358-6 18930529 PMC7135384

[29] Wang W, Dai JR, Liang YS. Apropos: factors impacting on progress towards elimination of transmission of schistosomiasis japonica in China. *Parasit Vectors*. 2014;7:408. doi: 10.1186/1756-3305-7-408 25175021 PMC4261779

[30] Zhu H, Yap P, Utzinger J, Jia TW, Li SZ, Huang XB, et al. Policy support and resources mobilization for the National Schistosomiasis Control Programme in the People’s Republic of China. *Adv Parasitol*. 2016;92:341–83. doi: 10.1016/bs.apar.2016.03.002 27137452 PMC7103126

[31] Yang Y, Zhou YB, Song XX, Li SZ, Zhong B, Wang TP, et al. Integrated control strategy of schistosomiasis in the People’s Republic of China: Projects involving agriculture, water conservancy, forestry, sanitation and environmental modification. *Adv Parasitol*. 2016;92:237–68. doi: 10.1016/bs.apar.2016.02.004 27137449

[32] Brattig NW, Bergquist R, Qian MB, Zhou XN, Utzinger J. Helminthiases in the People’s Republic of China: Status and prospects. *Acta Trop*. 2020;212:105670. doi: 10.1016/j.actatropica.2020.105670 32841589

[33] Lo NC, Gurarie D, Yoon N, Coulibaly JT, Bendavid E, Andrews JR, et al. Impact and cost-effectiveness of snail control to achieve disease control targets for schistosomiasis. *Proc Natl Acad Sci U S A*. 2018;115(4):E584–91. doi: 10.1073/pnas.1708729114 29301964 PMC5789907

[34] Sokolow SH, Wood CL, Jones IJ, Swartz SJ, Lopez M, Hsieh MH, et al. Global assessment of schistosomiasis control over the past century shows targeting the snail intermediate host works best. *PLoS Negl Trop Dis*. 2016;10(7):e0004794. doi: 10.1371/journal.pntd.0004794 27441556 PMC4956325

[35] Sokolow SH, Wood CL, Jones IJ, Lafferty KD, Kuris AM, Hsieh MH, et al. To reduce the global burden of human schistosomiasis, use ’old fashioned’ snail control. *Trends Parasitol*. 2018;34(1):23–40. doi: 10.1016/j.pt.2017.10.002 29126819 PMC5819334

[36] Qian C, Zhang Y, Zhang X, Yuan C, Gao Z, Yuan H, et al. Effectiveness of the new integrated strategy to control the transmission of *Schistosoma japonicum* in China: a systematic review and meta-analysis. *Parasite*. 2018;25:54. doi: 10.1051/parasite/2018058 30444486 PMC6238655

[37] Lei ZL, Zhang LJ, Xu ZM, Dang H, Xu J, Lv S, et al. Endemic status of schistosomiasis in People’s Republic of China in 2014. *Zhongguo Xue Xi Chong Bing Fang Zhi Za Zhi*. 2015;27(6):563–9. (in Chinese). 27097470

[38] Huang YX. Wetland protection and *Oncomelania hupensis* control. *Zhongguo Xue Xi Chong Bing Fang Zhi Za Zhi*. 2013;25(5):533–7. (in Chinese). 24490373

[39] Yang X, Zhang Y, Sun QX, Zhou JX, Zhou XN. SWOT analysis on snail control measures applied in the national schistosomiasis control programme in the People’s Republic of China. *Infect Dis Poverty*. 2019;8(1):13. doi: 10.1186/s40249-019-0521-0 30732636 PMC6367817

[40] Hao W, Yue-Ling X, Jia-Jing Z, Yang L, Yu-Ting Z, Ming-Xing X. Assessment of schistosomiasis transmission risk after flood damage in Wuhan City. *Zhongguo Xue Xi Chong Bing Fang Zhi Za Zhi*. 2018;30(4):410–4. (in Chinese). doi: 10.16250/j.32.1374.2018046 30350504

[41] Yang Y, Zheng SB, Yang Y, Cheng WT, Pan X, Dai QQ, et al. The Three Gorges Dam: Does the flooding time determine the distribution of schistosome-transmitting snails in the middle and lower reaches of the Yangtze River, China? *Int J Environ Res Public Health*. 2018;15(7):1304. 10.3390/ijerph15071304 PMC6069228 29933638

[42] Shi Y, Qiu J, Li R, Shen Q, Huang D. Identification of potential high-risk habitats within the transmission reach of Oncomelania hupensis after floods based on SAR techniques in a plane region in China. *Int J Environ Res Public Health*. 2017;14(9):986. 10.3390/ijerph14090986 PMC5615523 28867814

[43] Jia X, Bo Z, Yang L, Zi-Song W, Yi Z, Lin C. Impact of earthquake disaster on schistosomiasis transmission and emergency prevention and control in Sichuan Province. *Zhongguo Xue Xi Chong Bing Fang Zhi Za Zhi*. 2019;31(3):333–6. (in Chinese). doi: 10.16250/j.32.1374.2019072 31544421

[44] Liu Y, Xu BH, Chen L, Wu ZS, Xiao ZY, Wang CF, et al. Mid-term evaluation on schistosomiasis control effect in Lushan earthquake-stricken areas in Sichuan Province. *Zhongguo Xue Xi Chong Bing Fang Zhi Za Zhi*. 2013;25(5):467–72. (in Chinese). 24490354

[45] Shi YH, Wang BD. Schistosomiasis control effect in Mianyang City in ten years after Wenchuan earthquake. *Zhongguo Xue Xi Chong Bing Fang Zhi Za Zhi*. 2018;31(5):549–51. (in Chinese). doi: 10.16250/j.32.1374.2018069 31713393

[46] Ding C, Qiu Z, Zhu H. Multi-host transmission dynamics of schistosomiasis and its optimal control. *Math Biosci Eng*. 2015;12(5):983–1006. doi: 10.3934/mbe.2015.12.983 26280190

[47] Wang LD, Chen HG, Guo JG, Zeng XJ, Hong XL, Xiong JJ, et al. A strategy to control transmission of *Schistosoma japonicum* in China. *N Engl J Med*. 2009;360(2):121–8. doi: 10.1056/NEJMoa0800135 19129526

[48] Jiao-Jiao L. Endemic status and control of animal schistosomiasis in China. *Zhongguo Xue Xi Chong Bing Fang Zhi Za Zhi*. 2019;31(1):40–6. (in Chinese). doi: 10.16250/j.32.1374.2018313 31016922

[49] VAN Dorssen CF, Gordon CA, Li Y, Williams GM, Wang Y, Luo Z, et al. Rodents, goats and dogs—their potential roles in the transmission of schistosomiasis in China. *Parasitology*. 2017;144(12):1633–42. doi: 10.1017/S0031182017000907 28637527

[50] Liang S, Ponpetch K, Zhou Y, Guo J, Erko B, Stothard JR, et al. Diagnosis of *Schistosoma* infection in non-human animal hosts: A systematic review and meta-analysis. Preprints. 2021:2021050075. doi: 10.20944/preprints202105.0075.v1 PMC9116658 35522699

[51] Lv SB, Liu YW, Liu YM, Xu SG, Li YF, Yuan M, et al. Impact of “Elaphurus davidianus return home project” on the transmission of schistosomiasis in Poyang Lake areas. *Zhongguo Xue Xi Chong Bing Fang Zhi Za Zhi*. 2020;32(5):498–501. (in Chinese). doi: 10.16250/j.32.1374.2020039 33185061

[52] Ma XP, Zhao HM, Chen YC. The epidemiological status of schistosomiasis of wapitis in Shishou City of Hubei Province. *Chin J Vet Parasitol*. 2007;15(5):28–30.

[53] Yuan DS, Liu W, Zhang YS, Liu ZQ, Li Q. Investigation of epidemic situation of schistosomiasis in Gudao region of Changjiang in Shishou City. *Chin J Vet Parasitol*. 2006;14(1):20–2.

[54] Li SM, Deng WC, Cheng XH, He HB, Zhou YB, Zhou J, et al. Challenges and countermeasures of schistosomiasis control in Hunan Province in the new era. *Zhongguo Xue Xi Chong Bing Fang Zhi Za Zhi*. 2020;32(3):225–9. (in Chinese). doi: 10.16250/j.32.1374.2020051 32468782

[55] Secor WE. Early lessons from schistosomiasis mass drug administration programs. F1000Res. F1000 Faculty Rev 2015;4:1157. doi: 10.12688/f1000research.6826.1 26937275 PMC4752026

[56] Gray DJ, McManus DP, Li Y, Williams GM, Bergquist R, Ross AG. Schistosomiasis elimination: lessons from the past guide the future. *Lancet Infect Dis*. 2010;10(10):733–6. doi: 10.1016/S1473-3099(10)70099-2 20705513

[57] Zhou XN. Implementation of precision control to achieve the goal of schistosomiasis elimination in China. *Zhongguo Xue Xi Chong Bing Fang Zhi Za Zhi*. 2016;28(1):1–4. (in Chinese). 27356396

[58] Wang W, Yang K. Schistosomiasis and the global goals. *N Engl J Med*. 2020;382(16):1575–6. doi: 10.1056/NEJMc2002117 32294366

[59] Wang XY, He J, Juma S, Kabole F, Guo JG, Dai JR, et al. Efficacy of China-made praziquantel for treatment of schistosomiasis haematobium in Africa: A randomized controlled trial. *PLoS Negl Trop Dis*. 2019;13(4):e0007238. doi: 10.1371/journal.pntd.0007238 30969960 PMC6476521

[60] Yang K, Yang HT, Liang YS, Dai JR. Li Wei, Zhang JF, et al. A path analysis on China’s participation in global health governance: a case study of China Aid of Schistosomiasis Control in Zanzibar. *Zhongguo Xue Xi Chong Bing Fang Zhi Za Zhi*. 2019;31(1):14–8. (in Chinese). doi: 10.16250/j.32.1374.2019041 31016917

[61] Xu J, Yu Q, Tchuenté LA, Bergquist R, Sacko M, Utzinger J, et al. Enhancing collaboration between China and African countries for schistosomiasis control. *Lancet Infect Dis*. 2016;16(3):376–83. doi: 10.1016/S1473-3099(15)00360-6 26851829

[62] Xu J, Bergquist R, Qian YJ, Wang Q, Yu Q, Peeling R, et al. China-Africa and China-Asia collaboration on schistosomiasis control: A SWOT analysis. *Adv Parasitol*. 2016;92:435–66. doi: 10.1016/bs.apar.2016.02.005 27137455

